# Correcting visual acuity beyond 20/20 improves contour element detection and integration: A cautionary tale for studies of special populations

**DOI:** 10.1371/journal.pone.0310678

**Published:** 2024-09-26

**Authors:** Brian P. Keane, Steven M. Silverstein, Thomas V. Papathomas, Bart Krekelberg

**Affiliations:** 1 Center for Cognitive Science, Rutgers, The State University of New Jersey, Piscataway, NJ, United States of America; 2 University Behavioral Health Care, Rutgers, The State University of New Jersey, Piscataway, NJ, United States of America; 3 Department of Psychiatry, Robert Wood Johnson Medical School, Rutgers, The State University of New Jersey, Piscataway, NJ, United States of America; 4 Department of Psychiatry, University of Rochester Medical Center, University of Rochester, Rochester, NY, United States of America; 5 Department of Neuroscience, University of Rochester, Rochester, NY, United States of America; 6 Center for Visual Science, University of Rochester, Rochester, NY, United States of America; 7 Department of Brain & Cognitive Science, University of Rochester, Rochester, NY, United States of America; 8 Department of Ophthalmology, University of Rochester Medical Center, Rochester, NY, United States of America; 9 Department of Biomedical Engineering, Rutgers, The State University of New Jersey, Piscataway, NJ, United States of America; 10 Center for Molecular and Behavioral Neuroscience, Rutgers, The State University of New Jersey, Newark, NJ, United States of America; Federal University of Paraiba, BRAZIL

## Abstract

Contrary to popular lore, optimal visual acuity is typically better than 20/20. Could correcting acuity beyond 20/20 offer any benefit? An affirmative answer could present new confounds in studies of aging, development, psychiatric illness, neurodegenerative disorders, or any other population where refractive error might be more likely. An affirmative answer would also offer a novel explanation of inter-observer variability in visual performance. To address the question, we had individuals perform two well-studied visual tasks, once with 20/20 vision and once with optical correction, so that observers could see one line better on an eye chart. In the contour integration task, observers sought to identify the screen quadrant location of a sparsely defined (integrated) shape embedded in varying quantities of randomly oriented “noise” elements. In the collinear facilitation task, observers sought to detect a low-contrast element flanked by collinear or orthogonal high-contrast elements. In each case, displays were scaled in size to modulate element visibility and spatial frequency (4–12 cycles/deg). We found that improving acuity beyond 20/20 improved contour integration for the high spatial frequency displays. Although improving visual acuity did not affect collinear facilitation, it did improve detection of the central low-contrast target, especially at high spatial frequencies. These results, which were large in magnitude, suggest that optically correcting beyond 20/20 improves the detection and integration of contour elements, especially those that are smaller and of higher spatial frequency. Refractive blur within the normal range may confound special population studies, explain inter-observer differences, and meaningfully impact performance in low-visibility environments.

## 1. Introduction

**Fig 1:**
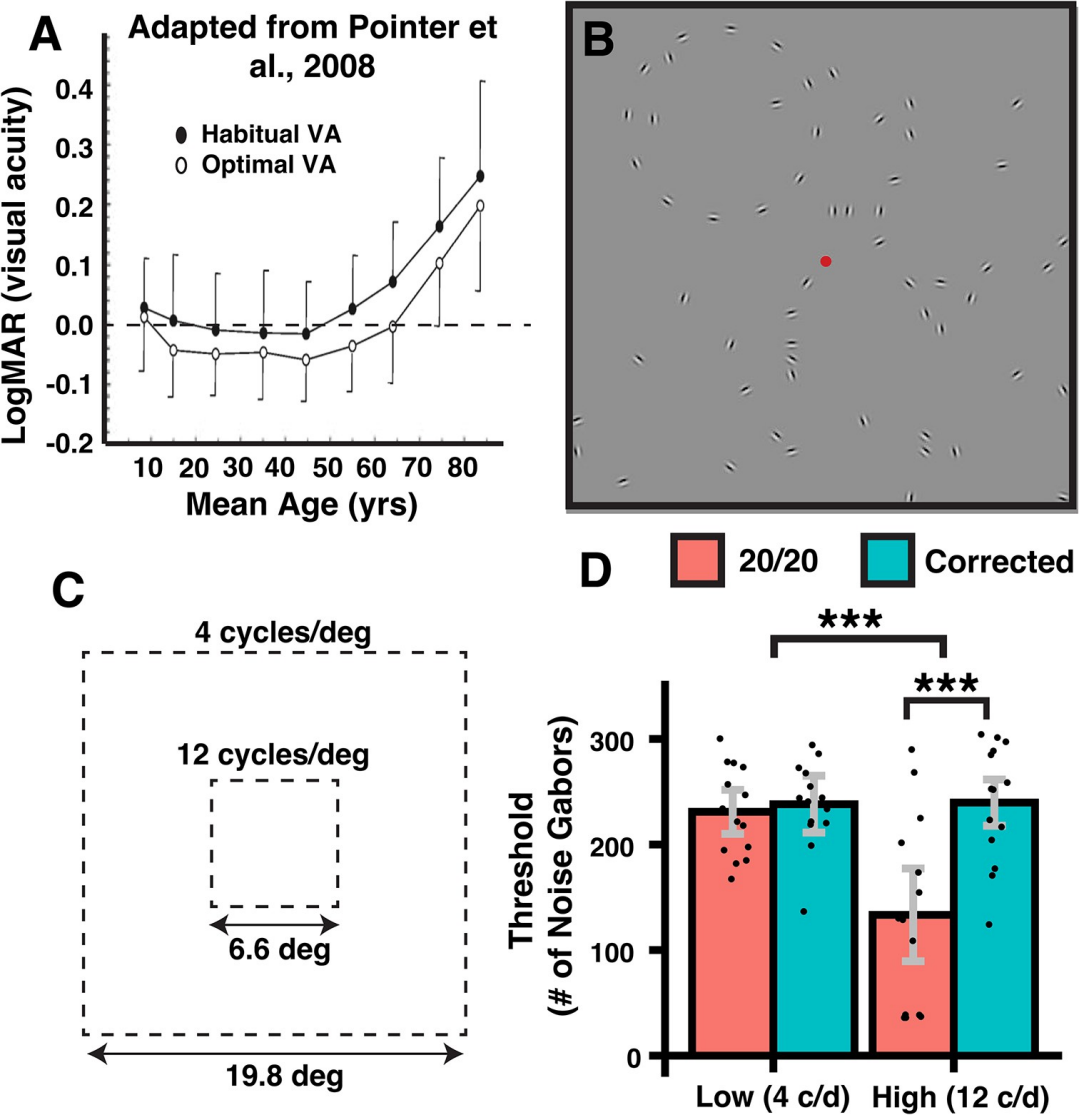
Motivation, stimulus, and results for the contour integration experiment. (A) Whereas habitual visual acuity is approximately 20/20 for the majority of adolescents and adults, acuity can typically be further improved via optical correction (see also [2]). This implies that it should be possible to optically improve not just acuity but also other aspects of visual performance among those with 20/20 vision (Figure adapted from [3]). (B) Observers in the contour integration experiment attempted to detect the screen quadrant location of a circular integrated target (shown here with a small number of randomly oriented “noise” elements). (C) The stimulus display was scaled larger or smaller to yield a lower and higher spatial frequency condition (4 or 12 cycles/deg). (C) Self-adjustable eyewear enabled observers to reach threshold accuracy (75%) under noisier conditions for the high spatial frequency condition. Errors equal ± 95% confidence intervals. *** p < .001.

Ever since Herman Snellen introduced his eye chart in 1862, optimal vision has often been thought to be 20/20, which corresponds to the ability to resolve gaps of 1 arc minute [1]. However, optimal visual acuity of healthy adult eyes is typically better than 20/20 [2, 3] (Fig 1A). Can improving acuity beyond 20/20 improve other aspects of vision? An affirmative answer would reveal a new confound for studies that do not match groups on refractive error and would offer a novel explanation for individual differences on vision tasks. It would also justify a re-evaluation of visual acuity requirements for consequential activities such as driving at night, flying aircraft, or conducting military or police operations in low-visibility environments.

To consider the relevance of optical correction within the normal range, we had observers with 20/20 vision perform two well-studied psychophysical tasks, once with 20/20 vision and once with vision improved beyond 20/20. In the contour integration task, observers sought to identify the quadrant location of a closed circular chain of oriented elements (“Gabors”) that appeared among a varying number of randomly oriented and positioned “noise” elements [4, 5]. In the collinear facilitation task, a central low-contrast target was presented between collinear or orthogonal high-contrast flankers, and observers determined on each trial whether that target was present or absent [6]. In each task, the entire display was scaled in size so that the elements were presented with a lower or higher spatial frequency (4 cycles/deg versus 10 or 12 cycles/deg). Scaling in this way allowed us to examine whether visual correction could become more relevant for less discernible features.

These two experimental paradigms have been of long-standing interest in special population studies because their biological substrates have been extensively investigated. Studies in psychophysics, electrophysiology, and single-unit recording have shown that contour integration and collinear facilitation are subserved by long-range horizontal excitatory connections between orientation-tuned spatial frequency filters in V1/V2 and by feedback from higher-order visual areas such as V4 [7–10]. These tasks have therefore been used to evaluate visual cortical functioning in aging [11], autism [12, 13], development [14], schizophrenia [15, 16], dyslexia [17], drug abuse [18], and amblyopia [19, 20], among other cases. Contrast sensitivity deficits—which can be revealed via the collinear facilitation task and which have a neural basis in the visual pathway [21]—have also been documented in a range of populations, including aging [22] and schizophrenia [23, 24]. In most cases, observers have “normal or corrected-to-normal” vision, which in its strictest definition, corresponds to having at least 20/20 vision. Here, we consider whether visual acuity confounds can arise when all subjects meet this requirement. An affirmative answer would show that inferences about visual cortical functioning in special population studies may often be premature.

In previous work, we compared healthy adults with 20/20 vision to those with better-than-20/20 vision (average logMAR difference = .12) on the experimental paradigms just described [25]. We found that the 20/20 group performed worse on the contour integration task and had worse contrast sensitivity on the collinear facilitation task, especially for small, high spatial frequency displays (scaled down in size). We hypothesized that the main effect and interaction in each task resulted from differences in refractive error. However, that study, being correlational, could not indicate the true origin of the deficit. Visual acuity has not just an optical component but also a cortical one, requiring a process of comparing a noisy incoming sensory representation to potentially 26 letter templates stored in long-term memory (assuming that most observers are English speaking and naïve to the Sloan optotypes) [26]. Eye movements are also relevant such that removing strategic micro eye movements via image stabilization can impair visual acuity by 1–2 lines on an eye chart (~0.15 logMAR units) [27]. Other factors, such as the integrity of the retina and optic nerve, and the pupillary reflex, can also impact acuity [28, 29]. What is needed, and what we provide here, is a simple but convincing demonstration that optical blur within the normal range can degrade visual perception for two commonly used and biologically well-described psychophysical tasks.

## 2. Materials and methods

### Data availability

The experimental data are available as supplemental material. The data file is in SPSS format (see S1 Data) and can be opened using the read.spss function in the foreign R library or in JASP. Definitions for the variables in the data file can be found in S1 File.

### Participants

The participants included 14 adults (8 females; age: *M* = 27.4 years, *SD* = 8.7, with age information lacking for one individual). Two participants were the co-authors (BK, BPK); all others were psychophysically inexperienced. None of these observers were dropped from the analysis and none discontinued the task due to fatigue or discomfort. The sample size was guided by the large effects uncovered in the aforementioned correlational study [25]. To be included in the study, observers needed to have at least 20/20 vision that was correctable by at least one line on an eye chart. We did not require any specific refractive error value before or after optical correction since many, if not most, special population studies exclude subjects based on subjective acuity rather than refractive error. The research followed the tenets of the Declaration of Helsinki and was approved by the Rutgers IRB. Accordingly, all participants provided informed written consent upon being apprised of the nature and possible consequences of the study.

### Optical correction

Acuity was established binocularly with a logarithmic (“ETDRS”) visual acuity chart (Precision Vision, LaSalle, Illinois) presented under fluorescent overhead lighting. Visual acuity values were expressed as logMAR units—the logarithm of the minimum angle of resolution (in arcmin) or the log10 of the Snellen fraction inverse, with 20/20 vision corresponding to 0.0 logMAR and with each line below it corresponding to downward steps of 0.1 logMAR. Passing a line required identifying at least three of the five letters on a line, and an observer’s acuity corresponded to the lowest line at which the observer was able to achieve that threshold. Note that, compared to Snellen charts, ETDRS test charts have better test-retest reliability and a lower floor for performance (20/10 rather than 20/16) [30, 31]. Note also that letter-based acuity charts (including Snellen) are routinely used in many scientific studies that critically depend on visual acuity, such as those that examine eye movements, spatial vision, and visual plasticity [27, 32–34].

Visual acuity estimates were obtained with the chart-recommended viewing distance of 2 meters. An advantage of this chart is that its estimates are robust and apply to a variety of distances, including those employed in our study (.88 meters and 1.82 meters) [35]. Note that our results would likely be the same if we had used a more standard ETDRS viewing distance of 4 meters. This is so because: i) visual acuity, on average, does not change between 0.5 and 7.5 meters [35], ii) accommodation response is small (~0.5 D) and has been empirically been shown to be almost identical for distances ranging from 1 meter to 8 meters [35], and iii) our participants were young and unlikely to have issues with lens accommodation. It should also be pointed out that severe hyperopia is unlikely in our study because the condition is uncommon in young adults [36] and because participants were able to reach 20/20 subjective acuity without optical correction.

Visual acuity was corrected via Adlens Emergensee glasses (purchase date: July 2013), which rely on Alvarez Dual Lens technology. The glasses consisted of a pair of slender lenses for each eye, with each lens having a wavefront curvature. By turning a knob for each eye, observers could slide the lenses laterally across each other to adjust the overall focal length. Twelve observers wore such glasses to improve their acuity by at least one line on the eye chart. One additional observer had prescription glasses to improve their vision, and another observer used the adjustable glasses to achieve both 20/20 and 20/16 vision. When using the adjustable glasses, observers covered one eye with an eye occluder and adjusted a knob on the side of the glasses until they could see the line below the lowest line they could see without the glasses as clearly as possible. This process was repeated for the opposite eye so that stimulus clarity would be improved binocularly. The binocular visual acuity before acuity correction was 20/20 (n = 12) or 20/16 (n = 2) (average logMAR = -0.01); the binocular acuity after correction was 20/16 (n = 12) or 20/12.5 (n = 2) (average logMAR = -.12). For expository purposes, we refer to the two visual acuity conditions as “20/20” and “corrected”, although it should be borne in mind that observers could likely have been corrected even further with specialized optometric equipment (see Discussion).

### Apparatus

The apparatus was the same as in our previous study [25]. Participants viewed stimuli from a chin rest on a 21” CRT monitor, which had a resolution of 1024x768 and a frame rate of 100 Hz noninterlaced. Lookup table values for the monitor were linearized with Psychophysics Toolbox [37] and calibrated on a regular basis with a Minolta CS-100 photometer (Konica Minolta Sensing Americas Inc, Ramsey, New Jersey). The room was darkened shortly before the tasks in order to minimize distractions from other details of the testing room and to be consistent with our earlier work. Participants fixated on a moderately bright screen (30 cd/m^2^) and thus observed the stimuli photopically. Note that the brightness of the screen made significant darkness adaptation unlikely. To the extent that there was darkness adaptation, its impact would be minimal since the ordering of the corrected and uncorrected conditions was counterbalanced across observers.

### Stimuli & procedure: General

Observers performed the contour integration task twice in succession, once with and once without optical correction. They also performed the collinear facilitation twice in succession, once with and once without optical correction. To counteract possible practice or fatigue effects, half of the observers always began each task with increased refractive error (“20/20”), and the remaining observers always began with reduced refractive error (“corrected”). The methods for both experiments have been previously described [25] but are repeated below.

### Stimulus & procedure: Contour integration

For the contour integration experiment, there was a lower spatial frequency (LSF) and a high spatial frequency (HSF) block of trials, which were counterbalanced across observers. Stimuli consisted of Gabor patches, which are oriented sinusoidal luminance gratings multiplied by a circular Gaussian:

$$G(x,y,\theta )=c\sin(2\pi f(x\sin\theta +\cos\theta ))\exp(-\frac{x^{2}+y^{2}}{2\sigma^{2}})$$

where (x,y) denotes the distance in degrees from the center of the element, *θ* is the element’s orientation (in deg), *f* is the peak spatial frequency of the element, and *c* is Michelson contrast. In the LSF block, Gabors had a sine phase (to create a balanced luminance profile), 95% contrast, a peak spatial frequency of 4 cycles/deg, and a Gaussian envelope SD (space constant) of 7.3 arcmin. Note that Gabor elements are commonly used in vision research since their luminance profile resembles the orientation-tuned spatial frequency filters found in early visual cortex and thus are thought to better activate populations in those regions [38]. The stimulus area (target + noise) subtended 19.9 deg on a side. The circular target (diameter = 7.4 deg) consisted of twelve equally spaced Gabors (spacing = 1.93 deg) and was positioned at a quadrant center with randomly added jitter (± 0.5 deg along each dimension). The target quadrant was randomly assigned on each trial and never contained a unique number of Gabors relative to neighboring quadrants. Noise Gabors never overlapped with each other or the target Gabors, and ranged in number from 36 to 464 depending on the staircase recommendation (see below). Stimuli in the HSF block were the same as the LSF block, except that the entire stimulus was scaled to one-third the retinal size (e.g., so that Gabors had a peak SF of 12 c/d). Scaling was achieved by shrinking the stimulus display and increasing the viewing distance from 87.6 cm to 181.5 cm. Note that modulating spatial frequency via viewing distance imposes little effect on contour integration from 3 to 24 cycles/deg [39]. On each trial, an array of oriented Gabors appeared for 1000 milliseconds (Fig 1B), after which participants saw a homogeneous gray screen with numbers 1 through 4 centered in each quadrant. Note that a 1 s presentation duration has been used successfully in past clinical studies [5, 40]. Observers were given an unlimited amount of time to identify the target quadrant number and did not receive feedback on response accuracy. There was no inter-trial interval.

Within a block, there were three randomly interleaved Bayesian adaptive “QUEST” staircases—30 trials per staircase—and each determined the number of noise patches needed to yield 75% accuracy [41]. The psychometric curves assumed a slope of 3 and an upper asymptote (1-delta) of .97. The three threshold estimates were averaged to produce one value per spatial frequency condition per observer. Thirty catch trials (without noise) also appeared randomly in each block to ensure that all observers were on task. Therefore, there were 240 non-practice trials in the experiment. Observers performed 20 practice trials that were of the same spatial frequency as the subsequent non-practice trials. The average duration for each of the two runs of this experiment (with or without correction), including instructions and practice, was 11.0 minutes.

#### Stimulus & procedure: Collinear facilitation

**Fig 2:**
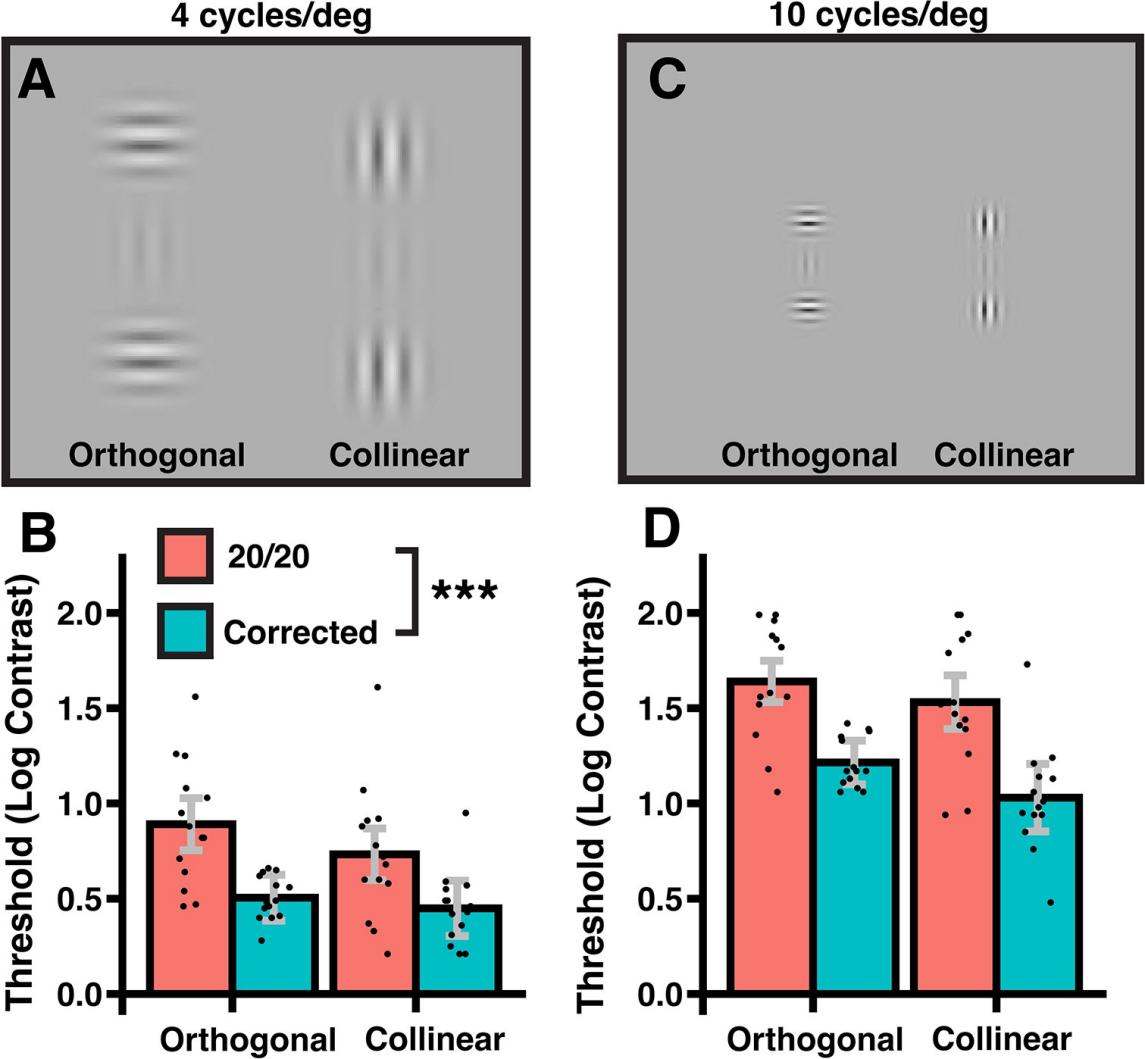
Stimulus and results for the collinear facilitation experiment. Orthogonal and collinear configurations and thresholds are shown for (A, B) a lower spatial frequency condition (4 cycles/deg) and (C, D) a high spatial frequency condition (10 cycles/deg). The task was to judge on each trial whether the central element was visible. Higher thresholds indicate that higher target contrasts were needed to see the target reliably. Optical correction improved thresholds for all conditions, but especially for those involving higher spatial frequency stimuli. Errors equal ± 95% confidence intervals. *** p < .001.

Stimuli in this experiment were viewed from a distance of 181.5 cm. Half of the collinear facilitation experiment consisted of low spatial frequency stimuli, and the other half consisted of high spatial frequency stimuli (Fig 2A and 2C). In the LSF trials, there were three vertically aligned Gabor elements centered on a mean gray background (45 cd/m^2^). The Gabors were rendered with the same formula as above; each had a sine phase, a peak spatial frequency of 4 cycles/deg, and a Gaussian envelope SD of 10.6 arcmin. The central Gabor was vertically oriented and separated from the flankers by 4 lambda (wavelength) center-to-center. The flanker orientations were either vertical (collinear) or horizontal (orthogonal), depending on the block. Note that edge-to-edge Gabor spacing at high contrast will be at least 15 arcmin and will not result in crowding on the foveola [42]. Therefore, detection thresholds of the central target across flanker conditions can provide an estimate of contrast sensitivity. Stimuli in the high spatial frequency trials were similar to the LSF trials, except that the entire stimulus was scaled to 40% of the retinal size (e.g., Gabors had a peak SF of 10 c/d). Similar to an earlier study [43], we increased the flanker contrast from 64% in the LSF block to 94% in the HSF block so that the latter would be easier to see. Flanker contrast differences within this range do not alter facilitation for lower SF stimuli [43].

Each trial began with a white fixation cross centered on a gray background. Immediately after initiating a trial, the observer saw a blank screen (400 ms), a three Gabor array (90 ms), and then another gray screen until a response was provided (present or absent). Note that the 90 ms presentation duration has been used successfully in past clinical studies to demonstrate a benefit of collinear flankers [44, 45]. After a response was entered, a gray screen appeared for 400 ms. We opted to present the stimulus on every trial rather than use a two-interval forced choice since qualitatively the same results arise in the two cases [44] and since the former allows for a shorter experiment. For each condition, 1-up, 3-down staircase determined the threshold, the amount of contrast needed to see the stimulus 79.4% of the time [46, 47]. Specifically, in the event of one incorrect response (miss), the contrast between the background and the central Gabor increased by 0.1 log units (26%); in the event of three consecutive correct responses (hit), the contrast decreased by the same amount. A decrease or increase of contrast preceded by a contrast change in the opposite direction was labeled a ‘reversal,’ and a block of trials terminated after seven reversals. Threshold for a condition was computed as the average contrast (in log units) for all the trials following the 4th reversal. (Averaging contrasts over all trials rather than just the reversal values improves threshold estimates [47]).

In each half of the experiment, there were two blocks corresponding to whether the flankers were orthogonal or collinear to the central target. The two block types were counterbalanced across observers, and so too was the SF order. Collinear facilitation was measured as the threshold in the orthogonal minus the collinear conditions, with larger (positive) differences reflecting more facilitation. The total number of non-practice trials was determined by how quickly subjects underwent 7 reversals in each of the four blocks; this corresponded to 204 trials (SD = 33 trials, range = [257, 158]) or about 40–60 trials per condition. Observers began each half of the experiment with 20 practice trials without flankers. The average duration for each of the two runs of this experiment (with or without correction), including instructions and practice, was 14.2 minutes.

### Analysis

For the contour integration task, groups were compared with two 2 (spatial frequency) by 2 (acuity) within-subjects type-III sums-of-squares analyses of variance (ANOVAs)—once for the catch trials (percent correct) and once for the non-catch trials (threshold averages). For the facilitation task, groups were compared with a 2 (spatial frequency) by 2 (flanker orientation) x 2 (acuity) within-subjects type-III sums-of-squares ANOVA. For the ANOVAs, we provide two effect sizes: $\eta_{p}^{2}$ which is most commonly reported in this context and *ω*^2^, which is less commonly reported but less biased [48]. Cohen’s d with Hedges’ correction was used to provide effect sizes of follow-up paired t-tests. All tests were parametric, assuming normality and independence of observations within subjects. Graphs show 95% confidence intervals, which are derived from the summarySE function in the Rmisc package in R, and which do not incorporate between-subject variance [49]. Analyses were conducted in SPSS version 29, except for the calculation of *ω*^2^, which was generated via JASP 0.19 [50].

## 3. Results

We investigated whether visual performance on contour integration or collinear facilitation changed as observers’ visual acuity was improved beyond 20/20 (Fig 1; see S1 Table).

In the contour integration task, for the catch trials, mean performance was consistently above 88% accuracy for each condition. There was a main effect of spatial frequency (*F*(1,13) = 6.054, *p* = .03, $\eta_{p}^{2}$ = .318, *ω*^2^ = .121) and no main effect of acuity (*F*(1,13) = 2.9, *p* = .11, $\eta_{p}^{2}$ = .183, *ω*^2^ = .121). There was a marginal two-way interaction (*F*(1,13) = 3.7, *p* = .08, $\eta_{p}^{2}$ = .223, *ω*^2^ = .067), indicating that small acuity changes might even impact the detection of solitary integrated shapes. In the non-catch trials, there were main effects of acuity (*F*(1,13) = 18.2, *p* < .001, $\eta_{p}^{2}$ = .583 *ω*^2^ = .253) and spatial frequency (*F*(1,13) = 8.3, $\eta_{p}^{2}$ = .391, *ω*^2^ = .164). The effect of acuity depended on SF (*F*(1,13) = 20.5, *p* < .001, $\eta_{p}^{2}$ = .628, *ω*^2^ = .222) such that visual correction did not alter performance at the low SF (*t*(13) = .7, *p* = .48, *Hedges’ g* = .19, 95% CI = (-.32, .68)), but it did help at the high SF (*t*(1,13) = 4.8, *p* < .001, *Hedges’ g* = 1.2, 95% CI = (.52, 1.86)). Finally, when we added age or sex (or both together) in the above ANOVAs, neither interacted with the other variables nor issued forth a main effect (all p>.05).

For the collinear facilitation experiment, there was an expected main effect of spatial frequency such that targets were less detectable (contrast thresholds were higher) for the scaled-down stimulus than for the larger display (*F*(1,13) = 814.0, *p* < .001, $\eta_{p}^{2}$ = .984, *ω*^2^ = .805) (Fig 2; see S1 Table). Contrast thresholds were also lower for collinear than orthogonal flankers (*F*(1,12) = 15.3, *p* = .002, $\eta_{p}^{2}$ = .540, *ω*^2^ = .105), exemplifying the classic collinear facilitation effect. There was also an acuity by spatial frequency interaction (*F*(1,13) = 7.2, *p* = .02, $\eta_{p}^{2}$ = .358, *ω*^2^ = .028). Follow-up t-tests (with thresholds averaged across orientation) showed that optical correction reduced contrast thresholds at the higher SF (*t*(13) = 4.8, *p* < .001, *Hedges’ g* = 1.21, 95% CI = (.53, 1.88) and, to a lesser extent, the lower SF (*t*(12) = 3.2, *p* = .006, *Hedges’ g* = .83, 95% CI = (.24, 1.41)). This two-way interaction dovetails with the contour integration results above and shows that smaller Gabors with finer-grained features are especially hard to detect for people with only 20/20 vision. No other interactions in the ANOVA reached significance (all *p* >.10). As above, when we added age or sex (or both) in the above ANOVAs, neither interacted with the other variables nor issued forth a main effect (all p>.05).

It may be questioned whether these effects depended on task sequence, that is, whether observers began each experiment with 20/20 vision or with optical correction (We thank a reviewer for suggesting this analysis). To consider this possibility, we re-ran the same ANOVAs as before but also included “sequence” as a between-subject factor (7 observers per group). In the contour integration task, there was a main effect of acuity and an acuity by spatial frequency interaction, as before (both *p*< = .001). Furthermore, there was no main effect of sequence and no interaction with sequence (all *p*>.12). In the collinear facilitation task, there continued to be a main effect of acuity and an acuity by spatial frequency interaction (both *p* < .01). However, here, sequence was relevant in exactly one way: it interacted with the acuity by spatial frequency interaction (*F*(1,12) = 5.0, *p* = .046, $\eta_{p}^{2}$ = .293, *ω*^2^ = .014). More specifically, the two-way interaction could be detected among those who began the task with 20/20 uncorrected vision (*F*(1,6) = 35.3, *p* = .001, $\eta_{p}^{2}$ = .855, *ω*^2^ = .064) but could not be detected among those who began with corrected vision (*F*(1,6) = .22, *p* = .65, $\eta_{p}^{2}$ = .036, *ω*^2^ < .001). Because this interaction was unexpected and just under the level of significance, and because there were only 7 subjects per group, the finding should be considered preliminary and in need of replication.

## 4. Discussion

We considered whether optically correcting visual acuity beyond 20/20 could benefit visual performance on contour integration or collinear facilitation tasks. In prior work, we hypothesized that there would be a benefit, especially at higher spatial frequencies [25]. The rationale was that removing slight amounts of refractive error might augment the detection of orientation, spatial frequency, position, or contrast, which in turn might strengthen integration processes that rely on these features. Consistent with our hypothesis, we found that improving acuity by about one line on an eye chart within the 20/20 range allowed individuals to tolerate the presentation of more noise Gabors in a high spatial frequency variant of a contour integration task and boost contrast sensitivity, especially for high SF stimuli. These effects (main effect and an acuity by spatial frequency interaction per task) were all large in magnitude ($\eta_{p}^{2}$ >.35) and in the predicted direction [25], and could survive statistical correction [51].

An objection may be that the glasses could have produced a placebo effect, improving confidence and overall performance. However, this would not explain why the glasses were more effective for the high versus low spatial frequency condition. Nor would it explain why our within-group differences closely matched the correlational results reported previously, where observers donned their own eyewear (if any). Finally, the placebo effect should not be assumed; the glasses could have alternatively led to complacency, causing observers to exert less effort in the expectation that the stimuli could be more easily observed.

### Implications for vision science, neuroscience, and society

Our results show that a common and easily fixed cause of imperfect acuity, refractive error, has a strong influence on two well-studied contour tasks even when acuity is within the normal range. Therefore, it is not sufficient to control for acuity simply by ensuring that all subjects have “normal or corrected-to-normal vision”; instead, *the groups must be matched on refractive error within the normal range*. Such matching can be done by testing all subjects with best corrected acuity (shortly after a refractive exam) or by statistically comparing groups on refraction values (derived from retinoscopy or an autorefractor). It is beyond the scope of our study to review all special population studies of vision that match or do not match groups on refractive error. However, failing to match on refractive error within the normal range could change the conclusions reached. For example, people with schizophrenia exhibit worse contrast sensitivity at higher spatial frequencies [23, 52, 53] but also more often have out-of-date prescriptions [54]. As another example, people of advanced age have impaired contour integration and contrast sensitivity at higher spatial frequencies [21, 55] but are also less likely to have appropriate eyewear [56]. In either situation, group differences might diminish or disappear entirely upon removing residual optic blur.

While matching groups on refractive error is a step in the right direction, it is probably not enough if the goal is to uncover the mechanisms responsible. As noted in the Introduction, visual acuity is influenced by a myriad of processes, including pupil size, lens opacity, retinal dopamine, integrity of the optic nerve, V1 surface area, eye movements, and perceptual learning [e.g., 27, 57–60]. Therefore, if groups are matched on refractive error but not on subjective visual acuity and if groups differ on either contour integration or contrast sensitivity, then it will be difficult to infer *why* the group differences emerge. As an example, subjective visual acuity improves in early childhood, worsens in older age [2, 26, 61], and is impaired in schizophrenia [62–65]. However, contour integration also improves in childhood [14], worsens in older age [11], and is impaired in schizophrenia [15]. Therefore, in each of these cases, integration differences could potentially be explained by any of the determinants of visual acuity.

At the same time, matching groups on visual acuity (and not just refractive error) must be done with care, as it could also generate misleading results if visual acuity is intrinsically linked to the condition of interest or if it shares mechanisms with the visual process of interest. For example, retinal hypo-dopaminergia worsens both contrast sensitivity and visual acuity [59], and may also be more common in chronic, stabilized schizophrenia patients [66]. In this context, matching patients and controls on visual acuity may remove some of the variance associated with the illness itself and may lead to an underestimate of contrast sensitivity deficits. Researchers will need to consider both approaches (matching and not matching on acuity) to reach a balanced and nuanced understanding of how a process is different in the population of interest.

Visual acuity confounds are probably more pervasive than what our results suggest. First, while our focus was on contour element detection and integration, small decrements in acuity could matter for *any* task in which fine-scale or low-visibility elements must be inspected. For example, crowding [67], orientation discrimination [68], and stereoacuity tasks [69] should all be reconsidered if the compared groups could realistically differ in visual acuity. In the neuroscience domain, the P1 component of the visual evoked potential has been linked to visual acuity differences [70], and this too might help explain why people with schizophrenia or people of advanced age have a longer latency and lower amplitude on this waveform [70, 71]. Second, we have defined “normal” vision as 20/20 or better but many studies have imposed more lenient visual acuity cut-offs. For example, in schizophrenia studies, the upper bound is commonly placed at 20/32 [24, 40, 72]. Thus, the opportunity for group differences in visual acuity is often larger than what we have assumed. Third, methods for measuring acuity are usually insufficiently precise, which could cause groups to *appear* matched on this variable when in reality they are not. As noted in our earlier work [25], Snellen charts yield noisier acuity estimates and often have lower bound limits of 20/16 or even 20/20 (e.g., Rosenbaum Pocket Eye Chart), making it difficult or impossible to discern whether groups are actually matched on acuity [31]. Adaptive staircase methods (Freiburg visual acuity test) may also yield noisier acuity estimates with an inadequate number of trials (>30) or response alternatives [73, 74] and thus may fail to pick up on true group differences (see Limitations below). Acuity estimates based on the number of letters correct are more precise than those based on the number of lines correct [75]. Going forward, we recommend that all studies include key details such as the lower cut-off for testing, the type of chart used, the method for scoring acuity, and whether groups are statistically matched on refractive error or subjective visual acuity.

Our results also point to an important way to increase statistical power: Whenever participant groups are already matched on acuity or whenever a good case can be made that the acuity differences are not essential to the group being studied, adding a visual acuity covariate will remove variance (noise) in the dependent variable and allow other group differences to stand out more clearly [76].

There are also implications that extend beyond neuroscience and vision science. Constable guidelines in the United Kingdom only require 6/6 binocular vision despite the importance of, for example, recognizing suspects in low-visibility environments [77]. The United States Federal Aviation Regulations only require 20/20 vision in each eye (www.faa.gov). Acuity standards for driving in the United States vary but can be as high as 20/120 (South Carolina) in the better eye (https://eyewiki.aao.org/Driving_Restrictions_per_State). This is concerning because recent clinical trials have shown that even a 0.5 diopter refractive error—roughly equivalent to a one-line difference on an eye chart relative to best-corrected acuity [78]—can worsen nighttime driving [79]. Tightening standards for vision may therefore improve public safety. Of course, the practical benefit of stricter acuity standards will need to be balanced with practical considerations (transportation availability) and will need to be confirmed in more ecological contexts.

### Are these results novel or surprising?

Our results have serious implications, but are they novel? For our literature search of contour integration (PubMed title/abstract keywords: “contour integration” AND refract*; search date = 8/05/24), the most relevant article showed that treatment-based visual acuity improvements in amblyopia was associated with improvements in contour integration [80]. However, acuity after correction in that study was approximately 20/20, making it unclear whether performance would improve even more with further reductions in refractive error. For collinear facilitation and contrast sensitivity, past work has shown that ~0.5 diopters of optical blur can worsen contrast sensitivity, especially at higher spatial frequencies [81–83] (PubMed abstract/title keywords: “contrast sensitivity” OR “collinear facilitation”) AND refract*; 1,258 retrieved search items retrieved; search date = 8/05/24). However, these studies either did not measure subjective visual acuity or they did not specify whether acuity was at least 20/20 in the presence of dioptric blur. Others have shown that worsening vision from 20/16 to 20/20 via spherical lenses numerically worsened contrast sensitivity, especially at higher spatial frequencies (range: 1.5 to 18 cycles/deg) [84]. Unfortunately, it was never reported whether the effect was statistically significant. Another study found similar effects for letter contrast sensitivity, but they did not report whether subjects had at least 20/20 vision after blur was induced [85]. Therefore, to our knowledge, the current study may be the first to directly show that optical manipulations *within the 20/20 range* can impact contrast sensitivity or contour integration.

But aren’t these results already obvious? Visual acuity is important for vision, and so of course increasing refractive error will worsen visual performance. Our response is that, for many researchers, this result is *not* obvious. For instance, the ICD-9 CM and the International Council of Ophthalmology [86] have placed the upper bound of “normal” vision at 20/25 (logMAR = .1) [see also Fig 7 of 87]. Many special population studies implicitly assume that the result is not obvious by not matching groups on refractive error within the normal range. As described above, visual acuity charts are often truncated, failing to include measurement levels of 20/10 or 20/12.5, implying again that these differences are unimportant.

### Limitations and future directions

A limitation of our study is that we optically corrected vision not by way of an optometrist but by self-adjustable corrective eyewear. However, the fact that vision can be corrected without any specialized equipment indicates that this improvement is attainable in everyday viewing circumstances. Moreover, an optometrist, having access to more advanced instrumentation (e.g., wavefront aberrometry), would likely have improved acuity to a greater extent than what we were able to (e.g., because of more precise spherical correction or correction for astigmatism or higher-order aberrations). Therefore, our results probably underestimate the benefits of transitioning to optimal vision from 20/20 vision. Likewise, we employed a commonly used and accepted method for measuring acuity—line-by-line ETDRS testing; however, our results may have been even stronger if we had measured acuity more precisely via letter-by-letter ETDRS testing or via certain variants of the Freiburg visual acuity test (e.g., more trials, more response alternatives, finer screen resolution) [73–75]. Future studies that seek to more powerfully demonstrate the functional importance of small amounts of refractive error would benefit from leveraging these more precise methods. It would also be worth knowing—via a standard clinical refraction exam—how much refractive error changed from our adjustable glasses and to what extent refractive error was still present in the “corrected” condition. Additionally, most of our observers initially had uncorrected 20/20 vision and so were likely adapted to optic blur [88]. Studies could consider adding defocus to those who already have optimal correction, to determine whether similar effect sizes might emerge in the absence of adaptation. Finally, we believe that refractive error confounds will arise when comparing groups for other types of visual processing (stereopsis, crowding, and so on), but this assertion will need to be verified experimentally.

## Supporting information

S1 Data Raw data. These data (in.sav format) were used to generate the results reported above.
(SAV)

S1 File Variable definitions. The variables of S1 Data are defined in this file.
(DOCX)

S1 Table Statisticsshown in bar graphs. This table provides means and 95% confidence intervals that were used to generate Figs 1B, 2B, and 2D.
(XLSX)
