# Supplementary material for: Correcting visual acuity beyond 20/20 improves contour element detection and integration: A cautionary tale for studies of special populations
Source: PLoS One. 2024 Sep 26;19(9):e0310678. doi: 10.1371/journal.pone.0310678 (PMC11426532; doi:10.1371/journal.pone.0310678)
Supplement: S1 File — The variables of S1 Data are defined in this file. (DOCX) [file pone.0310678.s002.docx]

**Variable Name: Definition**

SubID: Subject ID

BestVisFirst: Whether or not the subject was tested with corrected vision first in the sequence (1=yes, 0=no)

Age: Age in years

Sex: Sex of the participant

VA_good_both: visual acuity with correction, both eyes

VA_good_left: visual acuity with correction, left eye

VA_good_right: visual acuity with correction, right eye

VA_bad_both: visual acuity without correction, both eyes

VA_bad_left: visual acuity without correction, left eye

VA_bad_right: visual acuity without correction, right eye

CI_good_low: contour integration threshold, corrected acuity, low spatial frequency

CI_good_high_adj: contour integration threshold, corrected acuity, high spatial frequency

CI_bad_low: contour integration threshold, uncorrected acuity, low spatial frequency

CI_bad_high: contour integration threshold, uncorrected acuity, high spatial frequency

CI_good_low_catch: contour integration catch trial proportion correct, corrected acuity, low spatial frequency

CI_good_high_catch: contour integration catch trial proportion correct, corrected acuity, high spatial frequency

CI_bad_low_catch: contour integration catch trial proportion correct, uncorrected acuity, low spatial frequency

CI_bad_high_catch: contour integration catch trial proportion correct, uncorrected acuity, high spatial frequency

CF_good_logLowOrth: collinear facilitation, corrected acuity, log threshold, low spatial frequency, orthogonal flankers

CF_good_logLowCol: collinear facilitation, corrected acuity, log threshold, low spatial frequency, collinear flankers

CF_good_logHighOrth: collinear facilitation, corrected acuity, log threshold, high spatial frequency, orthogonal flankers

CF_good_logHighCol: collinear facilitation, corrected acuity, log threshold, high spatial frequency, collinear flankers

CF_bad_logLowOrth: collinear facilitation, uncorrected acuity, log threshold, low spatial frequency, orthogonal flankers

CF_bad_logLowCol: collinear facilitation, uncorrected acuity, log threshold, low spatial frequency, collinear flankers

CF_bad_logHighOrth: collinear facilitation, uncorrected acuity, log threshold, high spatial frequency, orthogonal flankers

CF_bad_logHighCol: collinear facilitation, uncorrected acuity, log threshold, high spatial frequency, collinear flankers

CF_good_log_Av: collinear facilitation, corrected acuity, log threshold, averaged across orientation and spatial frequency

CF_bad_log_Av: collinear facilitation, uncorrected acuity, log threshold, averaged across orientation and spatial frequency

CF_bad_minus_good_log_Av: collinear facilitation, log threshold, difference of averages between the uncorrected and uncorrected

CF_good_logHigh_Av: collinear facilitation, corrected acuity, log threshold, high spatial frequency, averaged across orientation

CF_bad_logHigh_Av: collinear facilitation, uncorrected acuity, log threshold, high spatial frequency, averaged across orientation

CF_good_logLow_Av: collinear facilitation, corrected acuity, log threshold, low spatial frequency, averaged across orientation

CF_bad_logLow_Av: collinear facilitation, uncorrected acuity, log threshold, low spatial frequency, averaged across orientation
